# The complexity of measuring reliability in learning tasks: An illustration using the Alternating Serial Reaction Time Task

**DOI:** 10.3758/s13428-022-02038-5

**Published:** 2023-01-05

**Authors:** Bence C. Farkas, Attila Krajcsi, Karolina Janacsek, Dezso Nemeth

**Affiliations:** 1 Université Paris-Saclay, UVSQ, Inserm, CESP, 94807 Villejuif, France; 2 Institut du Psychotraumatisme de l’Enfant et de l’Adolescent, Conseil Départemental Yvelines et Hauts-de-Seine, CH Versailles, 78000 Versailles, France; 3 Centre de recherche en épidémiologie et en santé des populations, Inserm U1018, Université Paris-Saclay, Université Versailles Saint-Quentin, Paris, France; 4 Department of Cognitive Psychology, Institute of Psychology, ELTE Eötvös Loránd University, Izabella utca 46, Budapest, H-1064 Hungary; 5 Centre for Thinking and Learning, Institute for Lifecourse Development, School of Human Sciences, Faculty of Education, Health and Human Sciences, University of Greenwich, Old Royal Naval College, Park Row, 150 Dreadnought, London, SE10 9LS UK; 6 Institute of Psychology, ELTE Eötvös Loránd University, Izabella utca 46, Budapest, H-1064 Hungary; 7 Brain, Memory and Language Research Group, Institute of Cognitive Neuroscience and Psychology, Research Centre for Natural Sciences, Magyar tudósok körútja 2., H, Budapest, –1117 Hungary; 8 Lyon Neuroscience Research Center (CRNL), INSERM U1028, CNRS UMR5292, Université de Lyon 1, Université de Lyon, Lyon, France

**Keywords:** Reliability, Procedural memory, Alternating Serial Reaction Time Task, Sequence learning, Statistical learning, Cronbach's alpha

## Abstract

Despite the fact that reliability estimation is crucial for robust inference, it is underutilized in neuroscience and cognitive psychology. Appreciating reliability can help researchers increase statistical power, effect sizes, and reproducibility, decrease the impact of measurement error, and inform methodological choices. However, accurately calculating reliability for many experimental learning tasks is challenging. In this study, we highlight a number of these issues, and estimate multiple metrics of internal consistency and split-half reliability of a widely used learning task on a large sample of 180 subjects. We show how pre-processing choices, task length, and sample size can affect reliability and its estimation. Our results show that the Alternating Serial Reaction Time Task has respectable reliability, especially when learning scores are calculated based on reaction times and two-stage averaging. We also show that a task length of 25 blocks can be sufficient to meet the usual thresholds for minimally acceptable reliability. We further illustrate how relying on a single point estimate of reliability can be misleading, and the calculation of multiple metrics, along with their uncertainties, can lead to a more complete characterization of the psychometric properties of tasks.

## Introduction

Although not extensively used in neuroscience and psychology, reliability assessment is extremely important, as low reliability negatively impacts standardized effect sizes, statistical power, and replicability. This issue is especially pertinent in correlational designs, which exploit natural variability in the measured constructs between different individuals (Dang et al., 2020; Enkavi et al., 2019; Hedge, Powell, & Sumner, 2018b; Miller & Ulrich, 2013). In such settings, the accurate measurement of the constructs is crucial for robust inference, as measurement error in the dependent and independent variables leads to attenuated effects, i.e., it generally biases regression slopes towards zero (Dang et al., 2020; Hedge, Powell, & Sumner, 2018b). For this reason, it is necessary to establish the reliability of experimental tasks as well, including in the domains of learning and memory (Green et al., 2016; West et al., 2018). In the mission of calculating the reliability of their experimental task, researchers are faced with several challenges. Here, we aim to illustrate several of these challenges and offer some tentative solutions.

The first one concerns the concept of reliability itself. Reliability refers to a measure's overall consistency, however there are a number of alternative ways to operationalize this general formulation, depending on our goals (Revelle & Condon, 2019). If the aim is to assess the capacity of a test to measure a temporally stable trait, then test–retest reliability is the most appropriate. If the aim is to assess the consistency between different raters observing the same phenomenon, inter-rater reliability is the most meaningful quantity. If the aim is to estimate how well different subsets of a larger tool measure the same construct, testing internal consistency is the most informative. Deciding which form of reliability to assess is already a difficult task. Although test–retest reliability is the most readily available and the most straightforward in interpretation, for tasks that measure highly time- and context-sensitive constructs, such as learning and acquisition, test–retest reliability assessments might not be feasible. In such cases, internal consistency might be the most informative option. However, the calculation of internal consistency metrics offers its own unique challenges. The most commonly used metrics, such as split-half reliability or Cronbach’s alpha require the task to be split into multiple components, between which to calculate some measure of agreement. Green et al. (2016) already explored various issues related to the choice of splitting in order to minimize the assumptions of internal reliability. However, their study focused on tasks that assess relatively stable and not highly time-sensitive cognitive constructs, such as the Stroop test for inhibition and a running span task for working memory. We believe that there are further factors to consider, when trying to estimate the reliabilities of online learning measures. By online measures, we refer to tasks and their metrics; which track learning as it unfolds in time, instead of relying on post-learning phase assessments (Siegelman et al., 2017). Such metrics, still implicitly or explicitly aim to measure a stable neurocognitive construct. Yet they tend to do so through constructing metrics that reflect some continuous improvement in performance on specific, carefully constructed stimulus sequences, that follow a particular regularity (for various examples, see Bogaerts et al., 2020; Gabriel et al., 2011; Hunt & Aslin, 2001; Nemeth, Janacsek, Londe, et al., 2010b). This makes them different in three important respects. One, as discussed in more detail below, offline consolidation and interference between sequences make the assessment of the same subject twice, in the exact same condition essentially impossible. Consequently, rendering test–retest reliability assessment unfeasible. Two, these tasks tend to be much longer, and the extraction of performance metrics requires many more trials. Three, performance is often assessed using relatively more complex techniques, such as difference scores between reaction times or accuracy to different kinds of stimuli (that differ, e.g., in their predictability), which creates numerous pre-processing choices that can heavily influence the estimated reliability.

A further issue is that most guidelines and practical tools for reliability assessment have been developed in the context of questionnaires. It is not obvious in all cases how one should adapt them to experimental tasks. Let's consider the definition of an 'item'. While identifying what a single 'item' is is rather obvious in the case of questionnaires, it is not that trivial for experimental tasks. Indeed, a large fraction of indices are constructed from multiple trials (such as difference scores in RT or accuracy); thus identifying a single trial as an item is clearly insufficient. Relatedly, questionnaires tend to have a single method of scoring, and thus one questionnaire corresponds to one metric in most scenarios. Again, that is not the case in experimental tasks, as multiple metrics can be constructed from the same task. Even when the psychometric properties of one metric have been established, they cannot be assumed to reflect other metrics from the same task. Thus, one must keep in mind that metrics (to be even more precise, metrics on particular samples), not tasks, are the appropriate unit of analysis for establishing psychometric properties. This paper explores these issues using a widely used sequence learning task as an example.

The alternating serial reaction time (ASRT) task is a visuo-motor probabilistic sequence learning task widely used for measuring (implicit) sequence/statistical learning, an aspect of procedural memory that is based on predictive processing (Kóbor et al., 2020; Nemeth, Janacsek, Balogh, et al., 2010a; Song et al., 2008; Takács et al., 2021). In this task, predetermined stimuli are interspersed with random ones (J. H. Howard & Howard, 1997; Janacsek et al., 2012), and this generative structure creates high-probability and low-probability stimulus triplets (see Methods). Subjects eventually develop a sensitivity to this difference and respond faster and more accurately to high-probability than low-probability triplets. This difference in reaction times or accuracy can then be taken as an index of learning performance. Robust learning is observed in the task, that has been shown to be stable for as long as 1 year (Kóbor et al., 2017), and be independent of explicit knowledge (Nemeth, Janacsek, & Fiser, 2013a; Vékony et al., 2021). The task has been employed to study a wide array of questions, ranging from the developmental trajectory of implicit and explicit learning (Nemeth, Janacsek, & Fiser, 2013a) to the effect of instruction on automatization and rewiring (Szegedi-Hallgató et al., 2017). It has been used with EEG (Horváth et al., 2021; Kóbor et al., 2019, 2021; Takács et al., 2021), non-invasive brain stimulation (Ambrus et al., 2020; Janacsek et al., 2015; Zavecz, Horváth, et al., 2020a), and both structural (Bennett et al., 2011) and functional MRI (Kóbor et al., 2022). It has also been employed to gain insight into both atypical neurocognitive development (Csábi et al., 2016; Nemeth, Janacsek, Balogh, et al., 2010a; Simor et al., 2017; Takács et al., 2018; Tóth-Fáber, Tárnok, Janacsek, et al., 2021a; Tóth-Fáber, Tárnok, Takács, et al., 2021b) and neurological or psychiatric disorders (Janacsek et al., 2018; Nemeth, Janacsek, Király, et al., 2013b; Unoka et al., 2017). Establishing the reliability of the commonly used metrics in this task is crucial to interpret these important results correctly. An influential recent paper by West et al. (2018) has highlighted a number of methodological issues with multiple widely used measures of procedural learning. Although not including the ASRT, their results suggested that different procedural learning tasks do not correlate highly with each other. Their test–retest and split-half reliabilities also fell well below minimally acceptable levels. If indeed the case, this severely limits the conclusions we can draw from results that rely on individual differences. This example highlights the importance of assessing reliability, even in experimental psychology settings.

The reliability of the ASRT has not yet been extensively studied. We are only aware of two previous studies reporting any kind of reliability coefficient for this task. Stark-Inbar et al. (2017) provided a test–retest reliability of learning scores of .46, whereas Buffington et al. (2021) arrived at a similarly sized split-half reliability of .42. One would not fault a researcher for concluding that the 'true' reliability of the task is in the .40 to .45 range. However, studies using the ASRT vary widely in multiple parameters that are known to affect reliability, such as task length, sample characteristics and the exact performance metric used. Therefore, it cannot be safely assumed that point estimates of reliability from one study generalize to another.

As we believe internal consistency estimates are the most informative in the case of online learning tasks, we calculate both simple split-half correlations as well as Cronbach’s alpha, for which we also provide analytic and bootstrap confidence intervals. We show how different pre-processing choices regarding the appropriate unit of splitting, and aggregation can lead to different reliability estimates. We further test the robustness of our estimates to alternative sampling of trials. Finally, we also explore how task length, and sample size affect reliability estimates in distinct ways. Briefly put, our own results suggest much higher reliabilities than previously reported, in the range of .70 to .80. This discrepancy is likely due to a number of factors, including our choice of Cronbach’s alpha as the primary reliability measure of interest, our relatively more sophisticated pre-processing pipeline, and the longer task. We hope this example highlights our primary message, that the calculation of a single estimate is insufficient to properly characterize the reliability of tasks and can be misleading, as there can be no single 'true' reliability of any task. A better approach is a more comprehensive assessment of multiple metrics, as well as explicit tests of their robustness to pre-processing choices and characteristics of the study samples.

## Methods

### Participants

One hundred and eighty subjects participated in the experiment (151 females, M_age_ = 21.61 years, SD_age_ = 4.14 years). All participants had normal or corrected-to-normal vision and none of them reported a history of any neurological and/or psychiatric condition. All participants provided written informed consent before enrolment and received course credits for taking part in the experiment. The study was approved by the United Ethical Review Committee for Research in Psychology (EPKEB) in Hungary (Approval number: 30/2012) and by the research ethics committee of Eötvös Loránd University, Budapest, Hungary. The study was conducted in accordance with the Declaration of Helsinki.

### The ASRT task

**Fig. 1:**
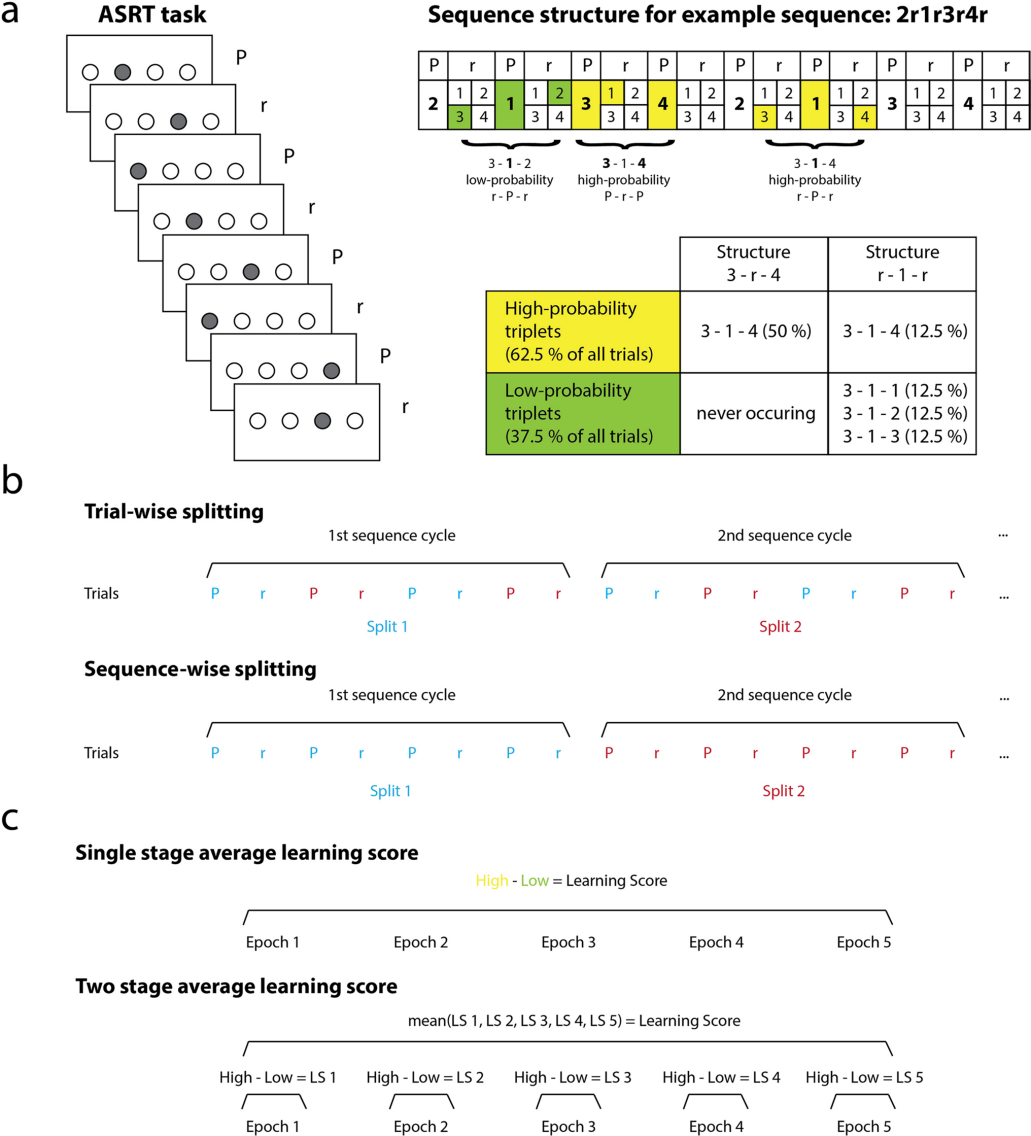
Task structure and reliability calculation procedures. **a** *Left* In the Alternating Serial Reaction Time (ASRT) task, a stimulus appeared in one of four horizontally arranged empty circles on the screen. The presentation of stimuli followed an eight-element sequence, within which predetermined (P) and random (r) elements alternated with each other. *Right* The alternating sequence in the ASRT task makes some runs of three consecutive stimuli (triplets) more frequent than others. High-probability triplets are indicated here with yellow and low-probability triplets with green. Importantly, high-probability triplets can result from two different arrangements of predetermined and random elements (P-r-P and r-P-r). **b** For reliability calculation, trials needed to be split into two halves. We employed two different ways of splitting. Trial-wise splitting meant that two successive trials (one pattern, one random) were considered as one unit, and assigned into a split (one denoted by the color *red*, the other by the color *blue* in this figure). Sequence-wise splitting meant that one full sequence cycle of eight trials (four pattern, four random) were considered as one unit, and assigned into a split. Both ensure, that there is an equal number of patterns and random trials in the two halves. **c** Learning scores can similarly be calculated in different ways. We employed two different ways of learning score calculation. Single-stage averaging meant that a single learning score per subject per split was computed, from all trials in a single stage, irrespectively of which epoch they belonged to. Two-stage averaging meant that in the first stage, five learning scores were calculated per subject per split, one per each epoch. In the second stage, these were then averaged

In our implementation of the ASRT task, a stimulus (a cartoon of a dog's head in our case) appeared in one of four horizontally arranged empty circles on the screen. Participants were instructed to press a corresponding key (Z, C, B, or M on a QWERTY keyboard) as quickly and accurately as possible when the stimulus occurred. Unbeknownst to the participants, the presentation of stimuli followed an eight-element sequence, within which predetermined (P) and random (r) elements alternated with each other (e.g., 2 − r − 1 − r − 3 − r − 4 − r; where numbers denote the four locations on the screen from left to right, and r's denote randomly chosen locations out of the four possible ones; see Fig. 1a). There were 24 permutations of the four possible spatial positions. However, because of the continuous presentation of the stimuli, for each participant, one of the six unique permutations of the four possible ASRT sequence variations was selected in a pseudorandom manner.

The alternating sequence in the ASRT task makes some instantiations of three successive stimuli (henceforth referred to as triplets) more frequent than others. In the example sequence given above, 2X1, 1X3, 3X4, and 4X2 (X indicates the middle element of the triplet) occurred often since the third elements could have either been a predefined or a random element (see Fig. 1a). At the same time, 1X2 and 4X3 occurred less frequently since the third element could have only been random. The former triplet types were labelled as "high-probability" triplets while the latter types were labelled as "low-probability" triplets. The third element of a high-probability triplet was more predictable from the first element of the triplet than in the case of low-probability triplets. For instance, in the example shown on Fig. 1a, Position 3 as the first element of a triplet is more likely (62.5%) to be followed by Position 4 as the third element, than either Position 1, 2, or 3 (12.5%, each). In accordance with this principle, each item was categorized as either the third element of a high- or a low-probability triplet, and the accuracy and reaction time (RT) of the response to this item were compared between the two categories. We excluded repetitions (e.g., 222) or trills (e.g., 232) from the analysis, as subjects can show pre-existing response tendencies, such as automatic facilitation, to these types of trials (Soetens et al., 2004). We also excluded trials with RTs lower than 100 ms and higher than 3 SDs above the subject specific mean RT, as these trials were likely to be errors due to inattention. For RT-derived learning scores, we only used correct trials, for accuracy-derived learning scores, naturally, both correct and incorrect trials were used.

One block of the ASRT task contained 85 trials. In each block, the eight-element sequence repeated ten times after five warm-up trials consisting only of random stimuli. The ASRT task was administered in three sessions, an initial learning session, a testing session after a 24-h delay, and a retesting session after a 1-year delay. Only the data from the learning session is analyzed in this study. Results from this sample have been previously reported in Fanuel et al. (2020); Kóbor et al. (2017); Quentin et al. (2021); and Török et al. (2017, 2021).

During the learning phase, which is the only phase we analyze here, the task consisted of nine epochs, each containing five blocks, equaling a total of 45 blocks.

Note that making participants aware that there is an underlying sequence to be discovered leads to the emergence of explicit knowledge of the alternating sequence structure, often termed higher-order sequence knowledge (Nemeth, Janacsek, & Fiser, 2013a). Whereas without such instructions, performance remains only driven by implicit sensitivity. We used the *Implicit* version of the task, without instructions.

### Reliability calculation – pre-processing choices

We choose to focus on internal consistency as we believe it is the most appropriate form of reliability metric for online learning, given that it minimizes interference from offline consolidation and practice effects (Kóbor et al., 2017; Szegedi-Hallgató et al., 2017). We estimate the internal consistency of the ASRT task by calculating Cronbach’s alpha. This coefficient ranges from 0 to 1, when applied to appropriate data, and higher values correspond to a greater degree of internal consistency (Streiner, 2003). The range of acceptable values depends highly on the context, generally, for research purposes, values between .65 and .9 are usually considered to be in the acceptable range, so that the test is coherent but not redundant (DeVellis, 2017; Streiner, 2003). Note, however, that alpha values, like all reliability estimates, should not be taken as entirely fixed properties of metrics, as they can depend on characteristics of the sample the test is administered to (Streiner, 2003). There are a number of intuitive interpretations of Cronbach’s alpha (McNeish, 2018):

1. It is the correlation of the scale/task of interest with another scale/task of the same length that intends to measure the same construct, with different items, taken from the same hypothetical pool of items
2. The square root of it is an estimate of the correlation between observed scores and true scores
3. It is the proportion of the variance of the scale/task that can be attributed to a common source
4. It is the average of all possible split-half reliabilities from the set of items

As it is most often used with questionnaires, it might come as a surprise, that alpha can be calculated meaningfully for a trial-based experimental task, however, it is entirely feasible (Green et al., 2016). One can conceive of alpha as a broad class of internal consistency coefficients, which can be calculated several different ways. For all results presented in this study, we used Eq. (5) of Green et al. (2016) to calculate alpha from two learning scores, calculated from two half splits of ASRT trials:

1 $$Cronbac{h}^{\prime } s\alpha =\frac{4{\sigma}_{half, half\prime }}{\sigma_{Task}^2}$$

where *σ*_*half*, *half*′_ is the covariance between the two scores, and ${\sigma}_{Task}^2$ is the variance of the sum of scores.

We also wanted to explore the effect of two pre-processing choices on our observed reliability estimates: The choice of splitting unit, and the choice of averaging level.

The choice of splitting unit refers to whether we consider a pair of trials (one pattern, one random), or a sequence cycle (eight trials, four pattern, four random) as a single unit during splitting to take into account the eight-element sequence structure of the task. Importantly, both ensure that there is an equal number of patterns and random trials in the two splits. We label the former method *trial-wise splitting*, and the latter *sequence-wise splitting* (Fig. 1b).

The choice of averaging level refers to whether we pull all trials from all epochs together to calculate a singular learning score in one step; or we first calculate a learning score for each epoch separately and then average them to obtain a singular learning score at the end. This latter option was considered because some previous ASRT studies calculated whole task learning scores for a given session this way (Tóth et al., 2017; Tóth-Fáber, Tárnok, Janacsek, et al., 2021a; Virag et al., 2015). We label the former method *single-stage average* and the latter *two-stage average* (Fig. 1c). Note, however, that the difference between the two procedures is also a question of validity, not just reliability. For example, if the pace of learning is strongly nonlinear, then the two procedures will be somewhat different, and two-stage averaging is likely to be more accurate.

These two options on the two choices gives us overall four possible ways of obtaining reliability measures. Our general algorithm for calculating these was thus the following:

1. Exclude trials below RT of 100 ms and above three times the SD of RTs for each subject, as well as trills and repetitions (and incorrect trials if RT-based score)
2. Split data into two half splits, either two halves containing equal number of pairs of trials (trial-wise splitting) or equal number of sequences of trials (sequence-wise splitting)
3. Calculate learning score as the difference in median RT or mean accuracy for high- and low-probability triplets in the two split halves separately, either in a single stage by pulling together trials from all epochs (single-stage averaging) at once, or in two stages by first separately calculating it in each epoch and then taking the average (two-stage averaging). Whatever the method, this results in two sets of learning scores, one learning score per subject per split.
4. Apply Eq. (1) to these two sets of scores

### Reliability calculation – reported metrics

We report the simple split-half correlation (i.e., the correlation between the sets of learning scores), but before applying Eq. (1) for the sake of completeness, and because some previous reliability estimates used this metric (Buffington et al., 2021; West et al., 2018). As explained above, Cronbach’s alpha goes beyond this metric by estimating the average split-half reliability of all possible splits, therefore, we also calculate alpha, and further recommend that researchers make use of it too. We also report two types of confidence intervals for alpha. The analytical one was calculated using the procedure outlined in Feldt et al. (1987). This procedure makes assumptions regarding the distribution of alpha, therefore, as an alternative procedure, a bootstrap confidence interval was also calculated. For this, we resampled participants from our dataset with replacement, 1000 times, and calculated alpha according to the algorithm described above for each sample. The resulting distribution of alphas is plotted for each type of alpha considered here. The .05 and the .95 percentiles of this distribution can be used to estimate the 95 % confidence interval.

In the initial analyses, we simply carry out the splitting in a sequential, even-odd manner: first splitting unit into the first split, second splitting unit into the second split, and so on. However, there is a possibility that this specific way of splitting the trials biases the obtained coefficients. To estimate the level of this bias, we also carried out a trial resampling analysis. We calculated alpha 1000 times according to the algorithm detailed above, and in each iteration, we simply split the task randomly into two halves, instead of proceeding sequentially. Comparing this distribution with the original, sequential estimates thus lets us see whether the original estimates are under- or overestimates. Thus, for each of our four types of split, we estimate reliability using four methods:

1. Simple sequential, even-odd splitting procedure, standard split-half correlation
2. Simple sequential even-odd splitting procedure, standard Cronbach’s alpha
3. Trial resampling distribution of Cronbach’s alphas
4. Bootstrap distribution of Cronbach’s alphas

### Task length and sample size

We also wanted to test how our observed alpha coefficients depended on task length and sample size. To investigate the effect of task length, we varied the number of successive blocks we included, from 1 to 45. For each task length, we calculate the *sequence-wise split, two-stage average* Cronbach’s alpha as well as the analytical 95% CI for both RT and accuracy learning scores.

To investigate the effect of sample size, we varied the number of subjects, from ten to the full 180, in steps of ten. Similarly to the task length analysis, for each sample size, we calculate the *sequence-wise split, two-stage average* Cronbach’s alpha as well as the analytical 95% CI for both RT and accuracy learning scores. However, given that there are multiple ways of choosing which subjects to sample for a given sample size, for each sample size, we resampled the included subjects 100 times and averaged both the alpha and its confidence interval across these 100 iterations.

## Results

We estimated the internal consistency of a widely used sequence learning task, the ASRT, using four different approaches. Simple split-half correlations, standard Cronbach’s alphas, the mean Cronbach’s alpha resulting from 1000 random splits of trials, and the mean Cronbach’s alpha resulting from a 1000 iteration bootstrap resampling of participants. We explored how these reliability estimates differed with different pre-processing choices, namely the choice of splitting unit (do we split pairs of trials, or longer sequences of trials?), and averaging level (do we aggregate data for the whole task in one stage, or in two stages, first in each epoch?).

### Reaction time-based learning scores

**Fig. 2:**
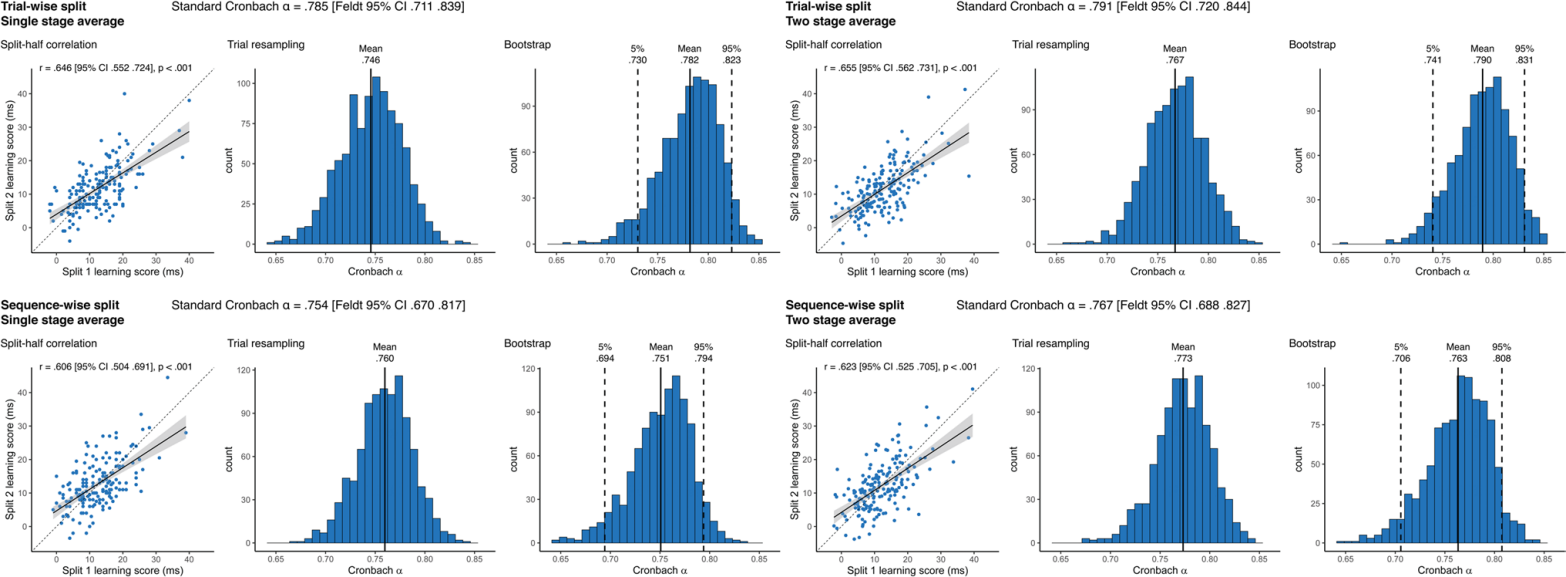
Reliability metrics for RT-derived learning scores. The four panels show the results of the four methods of reliability calculation that differ in pre-processing choices. In each panel, the Cronbach alpha on top of each panel shows the obtained alpha from the simple sequential assignment of trials, and its 95% CI calculated with Feldt's procedure. *Scatterplots* show learning scores the raw correlation between learning scores for the two splits, with one dot corresponding to one subject. Learning scores are in units of differences in reaction times for the two triplet types. The *trendline* shows linear fit, bands correspond to 95% CI. The *dashed line* shows the identity line. We also indicate the split-half Pearson's correlation and its *p* value, as well as 95% CI. Histograms show the results of the two permutation analyses, on the left, the distribution of Cronbach alphas resulting from trial resampling along with its mean, on the right, the bootstrapped distribution of Cronbach alphas, along with its mean, and the bootstrapped 95% CI values

The simple split-half correlations of RT learning scores were all statistically significant and ranged from .606 [95% CI .552 .724] for the *sequence-wise split, single-stage average* to .655 [95% CI .562 .731] for the *trial-wise split, two-stage average* metrics (Fig. 2). These corresponded to standard Cronbach’s alpha values ranging between .754 [analytical Feldt 95% CI .670 .817] and .791 [analytical Feldt 95% CI .720 .844], indicating respectable reliability (DeVellis, 2017). Interestingly, compared to these standard estimates trial resampling led to somewhat lower mean alphas for trial-wise, but not for sequence-wise splits. This suggests, that for RT learning scores, sequence-wise splitting might reduce the bias of only exploring standard sequential splits of trials. The mean value of the bootstrap distributions of alphas from the permutations agreed extremely well with the standard estimates, and the bootstrap CIs tended to be much smaller than the analytical ones. Moreover, both analytical and bootstrap CIs were somewhat larger for sequence-wise splits. Overall, averaging or splitting unit choices did not have a large effect on obtained reliability, although the two-stage average metrics were somewhat higher than the single stage average ones, suggesting that for RT learning scores, two-stage averaging might lead to more robust individual metrics.

### Accuracy-based learning scores

**Fig. 3:**
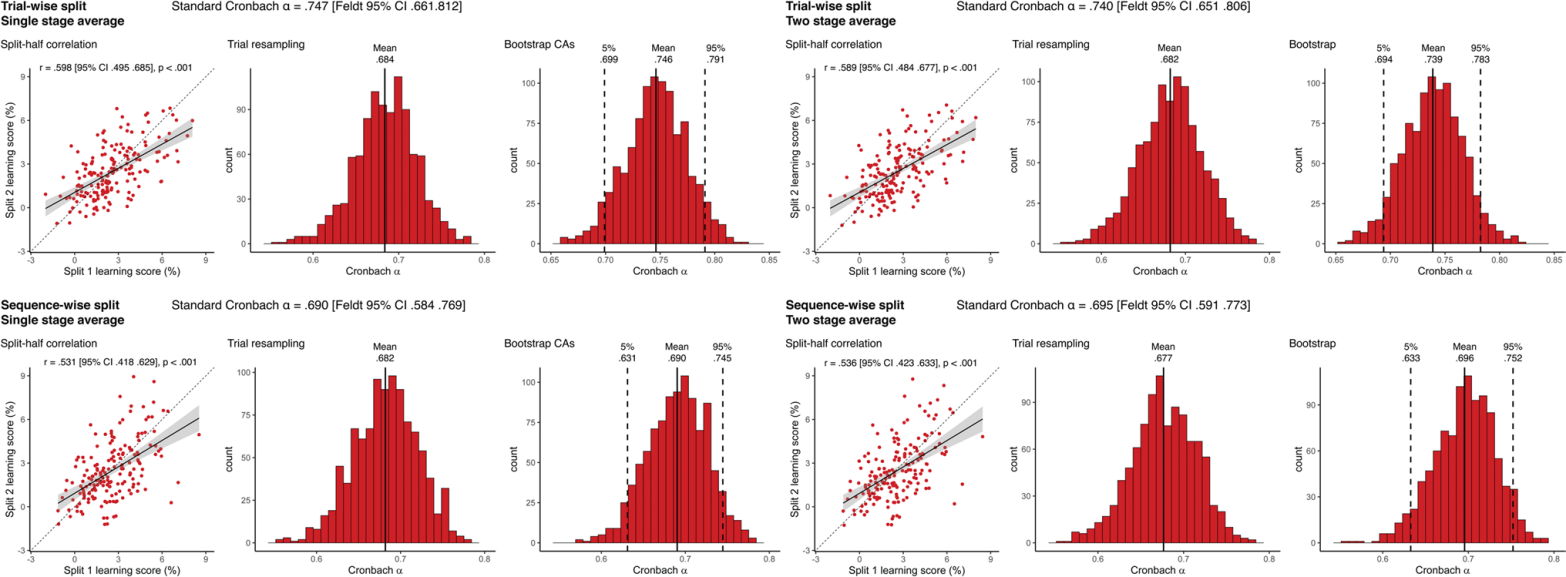
Reliability metrics for accuracy-derived learning scores. The four panels show the results of the four methods of reliability calculation that differ in pre-processing choices. In each panel the Cronbach alpha on top of each panel shows the obtained alpha from the simple sequential assignment of trials, and its 95% CI calculated with Feldt's procedure. *Scatterplots* show learning scores the raw correlation between learning scores for the two splits, one dot corresponding to one subject. Learning scores are in units of differences in reaction times for the two triplet types. The *trendline* shows linear fit, bands correspond to 95% CI. The *dashed line* shows the identity line. We also indicate the split-half Pearson's correlation and its *p* value, as well as 95% CI. Histograms show the results of the two permutation analyses, on the left, the distribution of Cronbach alphas resulting from trial resampling along with its mean, on the right, the bootstrapped distribution of Cronbach alphas, along with its mean, and the bootstrapped 95% CI values

The simple split-half correlations of accuracy learning scores were all statistically significant and ranged from .531 [95% CI .418 .629] for the *sequence-wise split, single-stage average* to .598 [95% CI .495 .685] for the *trial-wise split, single-stage average* metrics (Fig. 3). These corresponded to standard Cronbach’s alpha values ranging between .690 [analytical Feldt 95% CI .584 .769] and .747 [analytical Feldt 95% CI .661 .812], still respectable, but noticeably smaller than RT learning scores. Similarly to RT learning scores, compared to these standard estimates trial resampling led to somewhat lower mean alphas for trial-wise, but not for sequence-wise splits, again suggesting that the simple, sequential alphas might be overestimates. Again, the mean value of the bootstrap distributions of alphas from the permutations agreed extremely well with the standard estimates, and the bootstrap CIs tended to be much smaller than the analytical ones, and both types of CI were generally larger for sequence-wise splits. Averaging level did not influence the reliabilities strongly, contrary to RT, for these learning scores we did not observe higher alphas with two stage average calculation.

### Dissociation of RT- and accuracy-based learning scores

Interestingly and surprisingly, we found that accuracy and RT learning scores did not correlate strongly in our sample, *r* = .09 [95% CI – .057 .233]. As mentioned in the Introduction, measurement error in either the dependent or the independent variable can lead to attenuated correlational effects. Therefore, it is possible that the lack of a significant association is due to the measurement error in our learning scores. We can use our obtained reliability estimates to correct for this attenuation. However, using the procedure described in Charles (2005) for calculating corrected correlation coefficients and their 95% confidence sets, we still do not find a significant relationship, corrected *r* = .123 [95 % CI – .079 .314]. This at least partially explains the observed discrepancies between their reliabilities and recontextualizes previous studies that tended to utilize these two learning scores interchangeably (Takács et al., 2017, 2018).

### The effect of task length on reliability estimates

It is known that increasing task length and sample size impact reliability estimation in distinct ways (McNeish, 2018; Streiner, 2003). Increasing the length of tasks, and thus the number of trials / items, increases the size of the obtained reliability estimate, whereas increasing sample size improves its precision. To highlight the importance of these effects, we utilize our relatively large sample size and task length to investigate these effects in a practical context for the ASRT.

**Fig. 4:**
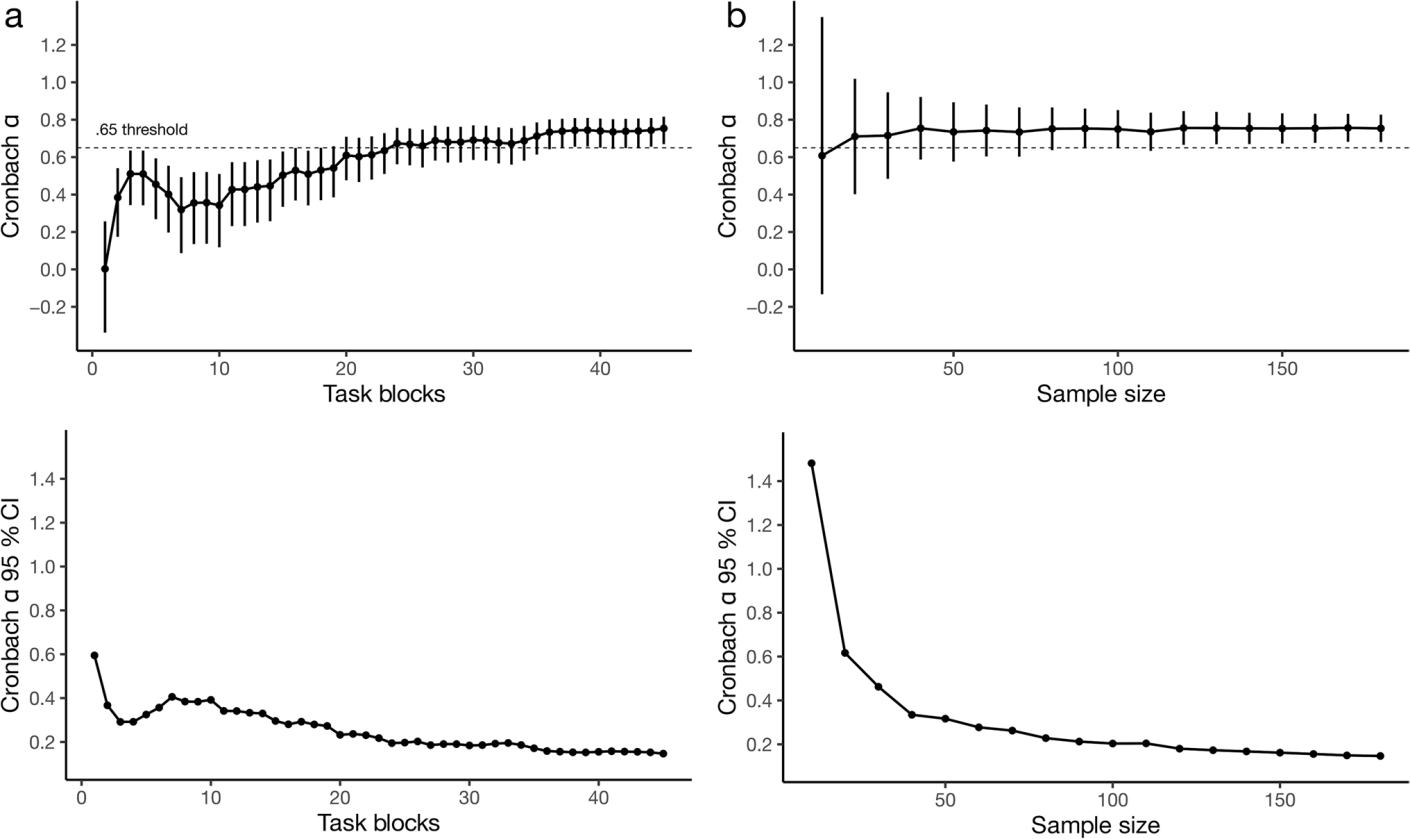
Permutation analyses of the effect of task length and sample size on the reliability of RT-derived learning scores. **a** We varied the number of blocks (max 45) to be included in the reliability calculation. The *top figure* shows the Cronbach alpha, and its Feldt 95% CI for each task length. The *dashed horizontal line* indicates the .65 level. The *bottom figure* shows the width of the 95% CI only. Increasing task length increases the point estimate of reliability, but has only a minor effect on its precision. **b** We varied the number of subjects (max 180) to be included in the reliability calculation. The *top figure* shows the mean Cronbach alpha across 100 random samples of subjects, and its Feldt 95% CI, for each sample size tested. The *dashed horizontal line* indicates the .65 level. The *bottom figure* shows the width of the 95% CI only. Increasing sample size has no effect on the point estimate of reliability, but increases its precision

**Fig. 5:**
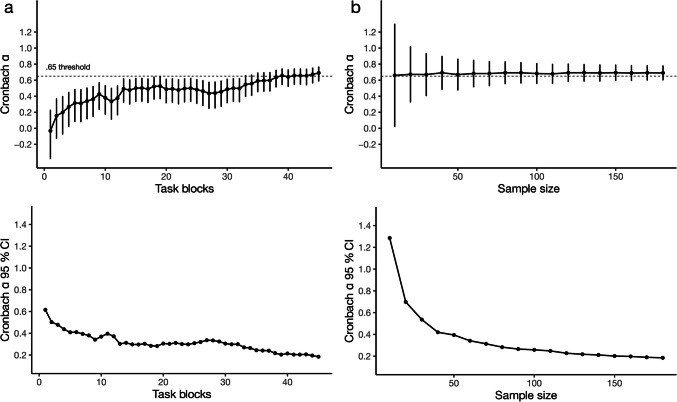
Permutation analyses of the effect of task length and sample size on the reliability of accuracy-derived learning scores. **a** We varied the number of blocks (max 45) to be included in the reliability calculation. The *top figure* shows the Cronbach alpha, and its Feldt 95% CI for each task length. The *dashed horizontal line* indicates the .65 level. The *bottom figure* shows the width of the 95% CI only. Increasing task length increases the point estimate of reliability, but has only a minor effect on its precision. **b** We varied the number of subjects (max 180) to be included in the reliability calculation. The *top figure* shows the mean Cronbach alpha across 100 random samples of subjects, and its Feldt 95% CI, for each sample size tested. The *dashed horizontal line* indicates the .65 level. The *bottom figure* shows the width of the 95% CI only. Increasing sample size has no effect on the point estimate of reliability, but increases its precision. The same pattern is observed as in the analysis or RT-derived learning scores, reported in the main text, the only difference is the overall lower alpha values

To investigate the scaling of reliability with task length, we calculated our *sequence-wise split, single-stage average* Cronbach’s alpha along with the analytical 95% CI for various all possible task lengths in our 45-block task. These simulation results confirm that reliability grows as task length increases (McNeish, 2018; Streiner, 2003). Interestingly, the marginal increase in reliability is not uniform across different lengths (Fig. 4). For RT learning scores, the reliability estimate reaches an alpha of .65, a threshold some have proposed to be minimally acceptable for research purposes (DeVellis, 2017; Streiner, 2003), around a task length of 25 blocks. This suggests that for RT learning scores a task length of 25 blocks might be sufficient to measure learning reliably, at least if other parameters are also similar to our case. For reaching the same threshold when using accuracy-based learning scores, a longer task length (around 40 blocks) seems necessary (Fig. 5). Irrespective of the learning measure of interest, depending on the study design and available resources for the study, longer versions of the task may be used to boost robustness of the estimates.

### The effect of sample size on reliability estimates

To investigate the scaling of reliability with sample size, we similarly varied sample sizes, from ten subjects to the maximum 180, in steps of ten, and calculated the *sequence-wise split, single-stage average* Cronbach’s alpha along with the analytical 95% CI for these sample sizes. Contrary to longer tasks, larger sample sizes do not primarily influence the point estimates of reliability. Rather, they increase the precision of these estimates. In other words, while more trials increase the reliability, more participants decrease the interval estimate of the reliability. We again observe a decreasing marginal gain, as the added precision of larger samples plateaus off around 100 subjects, at a 95% CI width of around 0.2. This analysis additionally suggests that our final sample size of 180 was likely adequate to estimate reliability. Similar approaches can be used by researchers as rudimentary post hoc checks on statistical power for reliability studies, which can accompany a priori power calculations. These might be necessary when there is very little data available to estimate likely values of Cronbach’s alpha, which are required for classical a priori calculations. To illustrate the issue using our case, using the methods reported in Bonett and Wright (2014), and aiming for a precision of .2 95% CI width, using two splits, we can estimate the required sample size for our study. However, if we base our estimate of alpha on the extant literature (Buffington et al., 2021; Stark-Inbar et al., 2017), that would likely put it somewhere around .45, which yields a corresponding sample size of 470. When we instead use the lowest alpha we obtained for RT, which was .754, the required sample is now a much more manageable 99 subjects. As this example highlights, due to the dearth of reported reliabilities in the literature, experimental psychologists will often not be in good positions to estimate likely reliability values, therefore such post hoc power checks as ours can be indeed rather useful.

## Discussion

We tested the reliability of RT- and accuracy-based learning scores, derived from the ASRT task on a large sample of 180 subjects. Calculating multiple split-half and Cronbach’s alpha metrics from multiple well-founded computations of learning scores, we found respectable reliability for all configurations tested. RT learning scores proved more robust than accuracy ones. Whether learning scores were calculated in a single step, or with a two-step procedure did not alter these estimates greatly. Accuracy-derived learning scores were, however, more robust when split halves were calculated at the trial level than at the sequence level. Moreover, a trial resampling procedure indicated that splitting trials at the sequence, instead of the trial level likely resulted in less biased estimates. Finally, we could also determine the scaling of reliability with both task length as well as sample size. We found that tasks with around 25 blocks of 85 trials each are likely sufficient to measure learning reliably, at least when using RT learning scores. The improvement in precision with increased sample sizes noticeably dropped off around 50 subjects, indicating that our sample size was likely enough or at least, that we could do no better, given the imprecision inherent in the task and its learning score metrics.

Besides the generally higher reliabilities of RT-based learning scores, RT- and accuracy-based learning scores were dissociable in another way. Namely, they did not correlate, which could explain the discrepancy between their reliabilities. We can only speculate about the lack of agreement between accuracy and RT learning measures. A possible methodological reason is that RT is an inherently continuous variable, but accuracy is indexed by an aggregate score of binary (0 – incorrect, 1 – correct) items, giving rise to some degree of information loss. Another more conceptual possibility is that these scores index quite different forms of learning. The usual model to capture the dependency between them is the drift diffusion model, which concisely explains speed–accuracy trade-offs resulting from varying parameters (such as decision thresholds) on an information accumulation mechanism (Forstmann et al., 2016). Although not including the ASRT or other statistical learning measures, a meta-analysis and simulation by Hedge, Powell, Bompas, et al. (2018a) suggests that such diffusion models can explain small or absent correlations between RT and accuracy costs in a range of experimental tasks. They show that whereas individual differences in accumulation rates produce a positive RT–accuracy correlation, individual differences in boundary separation produce a negative RT–accuracy correlation. Thus, if it is assumed that there are individual differences in both, then the correlation between effects in RTs and errors can be small or entirely absent. Further work using such models, as well as recent computational models of ASRT learning performance (Éltető et al., In press; Török et al., 2021) will be crucial in understanding the origins of RT- and accuracy-derived learning scores and exploring the factors affecting the presence or absence of correlations between the two.

We are aware of only two previous studies reporting the reliability of the ASRT task. Stark-Inbar et al. (2017) tested 123 subjects overall on three experimental tasks: a visuomotor adaptation task, the serial reaction time (SRT) and the ASRT tasks. For the ASRT, they tested 21 subjects in two sessions, separated by a 2–5-day interval. Group-level learning was evident in ASRT, and a significant test–retest correlation of .46 [95% CI 0.04, 0.74] was found between average learning scores of the two sessions, whereas no significant test–retest correlation emerged for the SRT learning scores. Note, however, that even though the authors tried to minimize consolidation effects by using a different mapping for the two sessions, the same underlying generative structure was employed in both timepoints, likely leading to some offline consolidation. Contrary to Stark-Inbar et al. (2017), we focused on internal consistency and split-half reliability, instead of test–retest reliability. Measuring the former is likely more appropriate for a task that aims to assess a crucially time-dependent process, such as online learning. Consolidation effects—that is, the changes that take place during the offline periods between subsequent learning sessions (Kóbor et al., 2017; Zavecz, Janacsek, et al., 2020b)—could significantly affect test–retest reliability. Furthermore, performing the same task with a different stimulus structure may potentially introduce proactive interference, whereby learning the second stimulus structure may be hindered by the initial knowledge (Hallgató et al., 2013; Szegedi-Hallgató et al., 2017), affecting test–retest reliability estimates. Nevertheless, we do not wish to discourage the assessment of stability entirely. We are merely suggesting that care has to be taken when interpreting test–retest reliabilities where the temporal stability of the test scores and/or underlying construct cannot be safely assumed. In such scenarios, test–retest reliability will reflect an unknown combination of within- and between-session noise. Moreover, between-session noise will likely be due to multiple sources, such as offline consolidation and interference effects, state-dependency, and potential long-term change in the cognitive construct itself. To illustrate, we calculated the Pearson correlation between the initial learning phase RT-derived learning scores (using two-stage averaging) and the learning scores obtained one year later, in a subset of 53 subjects, who were also assessed during that session. We obtained a correlation of *r* = .401 [.146 .606], *p* = .003. We can go one step further and try to use our internal consistency estimate to correct for within-session noise. Using Charles’s (2005) procedure, this leads to a corrected test–retest correlation of r = .523 [.166 .750], p < .001. These values are comparable to recently reported test–retest reliabilities of the ASRT and similar tasks (Arnon, 2020; Buffington et al., 2021; Stark-Inbar et al., 2017; West et al., 2018). This within-session noise corrected test–retest might yield an estimate that reflects between-session stability only. However, the problem is that we are still not in a position to disentangle the various sources of between-session noise. Thus, we believe in scenarios like this, the most appropriate ways of measuring and interpreting test–retest correlations are an open issue.

The second study, by Buffington et al. (2021), also administered multiple experimental tasks to a group of 99 subjects, including the ASRT task, and calculated split-half reliability. The Spearman–Brown split-half reliability of ASRT was found to be only a moderate .42 [95% CI 0.24, 0.57, calculated by us based on available information in their published paper]. Note, however, that in this study, instead of the most well-validated triplet-based learning scores (D. V. Howard et al., 2004; Nemeth, Janacsek, Király, et al., 2013b; Simor et al., 2019; Song et al., 2007) a different calculation of learning scores was employed that was based on a difference to individual pattern and random stimuli. Importantly, random trials can be either high or low-probability, and multiple studies have shown that participants are more sensitive to triplet-based probabilities than the alternating pattern-random structure, at least with one session of practice (D. V. Howard et al., 2004; Nemeth, Janacsek, & Fiser, 2013a; Song et al., 2007). Therefore, the learning score based on pattern-random difference likely underestimates the true learning that occurs in the task. Furthermore, unlike Buffington et al. (2021), we excluded trials that were repetitions (e.g., 222) or trills (e.g., 232), as subjects likely have pre-existing response tendencies to such trials (Soetens et al., 2004). This also possibly increased the robustness of our learning scores. Overall, the triplet-based learning scores we employ here are likely better suited to reliably measure learning in the ASRT task.

While not including the ASRT specifically, a study by West et al. (2018) estimated the reliability of multiple declarative (word list, dot location, immediate serial recall) and procedural memory tasks (SRT, Hebb serial order, contextual cueing) in a large sample of children. They found higher split-half reliability for declarative tests (ranging between 0.49 and 0.84) than for procedural tests (ranging between – 0.03 and 0.75). However, although procedural tasks showed lower reliability on average, this was mainly driven by the extremely low reliability of the contextual cueing and verbal SRT tasks. The non-verbal SRT task with a probabilistic sequence structure, which resembles the ASRT the most, in fact had a split-half reliability of .75 in the first session [95% CI 0.65, 0.83, calculated by us based on available information in their published paper], on par with the observed reliability of declarative tasks and our results. The lower split-half reliability of .49 on the second session [95% CI 0.31, 0.63, calculated by us based on available information in their published paper] was likely due to the almost ceiling level performance by the subjects, instead of a shortcoming of the task. Moreover, the low test–retest correlation of .21 [95% CI 0.00, 0.40, calculated by us based on available information in their published paper] of the non-verbal SRT task might be more due to the inappropriateness of assessing test–retest reliability for learning tasks, the ceiling level performance in the second session, as well as consolidation/proactive interference effects (see above). Overall, based on our current study and the study of West et al. (2018), non-verbal SRT tasks with probabilistic stimulus structures seem to have respectable internal consistency both in children and adults.

It is also interesting to compare these results to a recent study by Arnon (2020), that investigated the reliability of linguistic auditory, non-linguistic auditory and non-linguistic visual statistical learning tasks in adults and children. Similarly to the results summarized above, Arnon (2020) found relatively high internal consistency, but low test–retest reliability of the visual SL task, in both adults and children. Interestingly, the auditory SL tasks had much lower internal consistency, but higher test–retest reliability. Moreover, reliability was far lower in children, than in adults, hinting at important age differences not only in statistical learning, but the reliability of SL tasks themselves.

All of the above highlights the fact that a point estimate of a single reliability metric is often insufficient to fully characterize the properties of experimental tasks, as various, seemingly innocuous pre-processing and splitting choices can impact them in important ways. Psychometric properties of tests, such as their reliability, are not fixed properties of scales, independent of context (Streiner, 2003). Besides choices in the pre-processing of task scores and the calculation of reliability metrics, such estimates are often influenced by the sample that a given task is administered to as well. For example, clinical samples can be associated with altered reliability estimates (Caruso, 2000; Lakes, 2013). Given that a major line of procedural memory research, which makes extensive use of the ASRT and similar tasks, aims to relate it specifically to neurodevelopmental disorders (Ullman et al., 2020), it is crucial that internal consistency of these tasks are tested in such specific samples as well. These considerations further reinforce the need to report reliability coefficients and their uncertainties in published experimental psychology results, as relying on a few previously estimated values can be extremely misleading.

A common use of reliability is for the correction of measurement error, often termed correction for attenuation (Muchinsky, 1996; Revelle & Condon, 2019). When we are interested in the relationship between two latent constructs, either one or both being measured with an imperfect tool, we can use the reliability of the tools to obtain a better estimate of the true underlying correlation between the two constructs. For example, in Virag et al. (2015), we previously reported a negative correlation of *r* = – .420 between an executive function *z*-score measure and ASRT RT learning scores as evidence for competition between frontal lobe functions and implicit sequence learning. If we now take into account the reliability of ASRT we found here, we can calculate a corrected estimate of this correlation by dividing it with the square root of the reliability. In this case (using the *sequence-wise split, two stage average* reliability), this leads to a corrected correlation coefficient of r = $\frac{-.420}{\sqrt{.767}}$ = -.480. It is also possible to obtain confidence intervals for corrected correlation coefficients, although the way to do so is somewhat more complex, than for standard correlations (see Charles (2005) for one possible procedure). Thus, the estimation of reliability can be used to increase observed effect sizes by taking measurement error into account.

We aimed to highlight multiple obstacles in the reliability estimation of experimental tasks researchers are likely to encounter, using the Alternating Serial Reaction Time task as a concrete example. These challenges, our tentative solutions, and the concrete results pertaining to the ASRT are summarized in Table 1. Firstly, the choice of what form of reliability to estimate is already crucial, as not all forms are appropriate for all contexts. Test–retest reliability might not be feasible for estimating the reliability of online learning scores, as offline consolidation and interference effects can bias results. Indeed, we obtained larger reliability estimates using split-half and internal consistency forms, than previous studies reporting test–retest reliability (Stark-Inbar et al., 2017; West et al., 2018).

**Table 1:** Summary of the challenges of reliability estimation for online learning tasks that are discussed here. The general problem, our proposed recommendations, and their concrete illustration using the ASRT are shown

Challenge	Possible solution	Concrete example with the ASRT
Not all forms of reliability can be meaningfully evaluated in all contexts	Determine appropriate reliability forms	Interference and offline consolidation effects make test–retest reliability unfeasible for the ASRT. Rely on internal consistency and split-half reliability instead
Multiple performance metrics can be calculated from the same task, the reliabilities of which cannot be assumed to be equivalent	Estimate reliability for each metric separately	Accuracy and RT-based learning scores have distinct reliability profiles, with RT-based learning scores being somewhat more reliable Learning scores calculated using two-stage averaging are generally more reliable Triplet-based learning scores are more reliable, than pattern-random trial difference scores
Different pre-processing choices regarding splitting can lead to distinct reliability estimates	Investigate robustness of reliability estimation to splitting choices, e.g., by varying the units of splitting and carrying out trial-resampling	Splitting by sequences instead of trials leads to lower reliability, with more variance, but possibly less bias
Task length influences reliability estimation, with longer tasks being associated with higher reliability estimates. This needs to be taken into account when interpreting published reliability estimates and designing studies	Determine the scaling of reliability estimates with increasing task length	Threshold for 'minimally acceptable' reliability of .65 is met with a task length of around 25 blocks
Sample size influences reliability estimation, with larger samples being associated with more precise reliability estimates. This needs to be taken into account when interpreting published reliability estimates	Determine the scaling of the precision of reliability estimates with increasing sample size	Marginal gains in the precision of reliability estimates drop off noticeably around 50 subjects

Secondly, the possibility of calculating multiple performance metrics from the same task further complicates reliability estimation and interpretation, as reliabilities for one cognitive metric and one pre-processing procedure cannot be assumed to reflect other metrics and pre-processing choices. In the context of the ASRT, this issue manifests itself in multiple ways. We obtained much higher reliability with our triplet-based learning scores, compared with previous estimates that only used pattern-random difference scores (Buffington et al., 2021), which suggests that these might be better suited for robust measurement of individual learning performance. We also observed differences between accuracy and RT-based learning scores, with RT learning scores proving more reliable, and the two learning scores not correlating across individuals. This strongly suggests that these measures cannot be used interchangeably. A further difference emerged between single-stage and two-stage average learning scores, with the latter being somewhat more reliable, at least for RT learning scores.

Thirdly, in the case of split-half and internal consistency, it is also advisable for the robustness of obtained reliability estimates to be tested against alternative choices of splitting the task. Here, we explored whether the unit of splitting influenced results by comparing reliability estimates from trial-wise and sequence-wise splits. Splitting by sequences instead of trials leads to lower reliability estimates with more variance. However, our trial resampling procedure also indicated that the reliability of sequence-wise splits obtained from even-odd splitting agreed more with the distribution of reliabilities obtained from randomly reshuffling splitting units. Which suggests that these estimates might be less biased by even-odd splitting.

Finally, both the length of the task, and the sample size of the study are known to impact reliability estimation in distinct ways. Increasing task length primarily increases the size of obtained reliability, whereas increasing sample size primarily increases its precision. While establishing rigid thresholds is unfeasible and unadvisable, our analysis of these effects for the ASRT indicated that a length of 25 blocks can be sufficient to reach conventional minimally acceptable reliability thresholds for research, at least for RT learning scores. Longer task lengths should nevertheless be preferred as they result in even higher reliability. However, we are aware that such lengthy tasks might not be feasible in every scientific context. For example, in the case of child or clinical samples, they might lead to significantly more attentional lapses, outliers, larger dropout rates, and ironically, worse quality data. In such cases, these negative consequences should be weighed against higher reliability. We still recommend that at least a task length of 25 blocks should be achieved, if possible. We also showed that although precision grows with larger samples, the marginal increase in precision decreases, and shrinks to negligible levels after a sample size of around 50 subjects is reached. This suggests that future psychometric research of the ASRT should be carried out with at least 50 subjects.

We hope our exploration of these complexities has demonstrated not only the challenges, but also the value of reliability assessment of learning tasks. Having accurate information about the strengths and weaknesses of our instruments is a necessary first step in making informed research decisions. However, the estimation of reliability itself requires careful consideration as well, to identify and overcome common issues, including the types we highlighted here as well as many other ones. Researchers aiming to use learning tasks need to take these factors seriously, such that we can build a robust and reproducible science of learning and memory. The adoption of the approach of multi-metric reliability assessment we advocate here, will go a long way towards this goal.

## Data Availability

All data pertaining to this study are freely available on the OSF repository, at https://osf.io/9szk7/.

## Code availability

All code pertaining to this study are freely available on the OSF repository, at https://osf.io/9szk7/.
