# Correction: The complexity of measuring reliability in learning tasks: An illustration using the Alternating Serial Reaction Time Task

**DOI:** 10.3758/s13428-025-02670-x

**Published:** 2025-06-03

**Authors:** Bence C. Farkas, Attila Krajcsi, Karolina Janacsek, Dezso Nemeth

**Affiliations:** 1 Université Paris-Saclay, UVSQ, Inserm, CESP, 94807 Villejuif, France; 2 Institut du Psychotraumatisme de l’Enfant et de l’Adolescent, Conseil Départemental Yvelines et Hauts-de-Seine, CH Versailles, 78000 Versailles, France; 3 Centre de recherche en épidémiologie et en santé des populations, Inserm U1018, Université Paris-Saclay, Université Versailles Saint-Quentin, Paris, France; 4 Department of Cognitive Psychology, Institute of Psychology, ELTE Eötvös Loránd University, Izabella utca 46, Budapest, H- 1064 Hungary; 5 Centre for Thinking and Learning, Institute for Lifecourse Development, School of Human Sciences, Faculty of Education, Health and Human Sciences, University of Greenwich, Old Royal Naval College, Park Row, 150 Dreadnought, London, SE10 9LS UK; 6 Institute of Psychology, ELTE Eötvös Loránd University, Izabella utca 46, Budapest, H- 1064 Hungary; 7 Brain, Memory and Language Research Group, Institute of Cognitive Neuroscience and Psychology, Research Centre for Natural Sciences, Magyar tudósok körútja 2., H, Budapest, 1117 Hungary; 8 Lyon Neuroscience Research Center (CRNL), INSERM U1028, CNRS UMR5292, Université de Lyon 1, Université de Lyon, Lyon, France

**Correction: Behavior Research Methods (2023) 56:301–317**

10.3758/s13428-022-02038-5

The original online version of this article was revised: Figures 2 and 3 did not correspond to what was originally intended. The updated figures contain confidence intervals and bootstrap histograms. No changes were made to figure legends.

**Incorrect Figure 2**

**Fig. 2:**
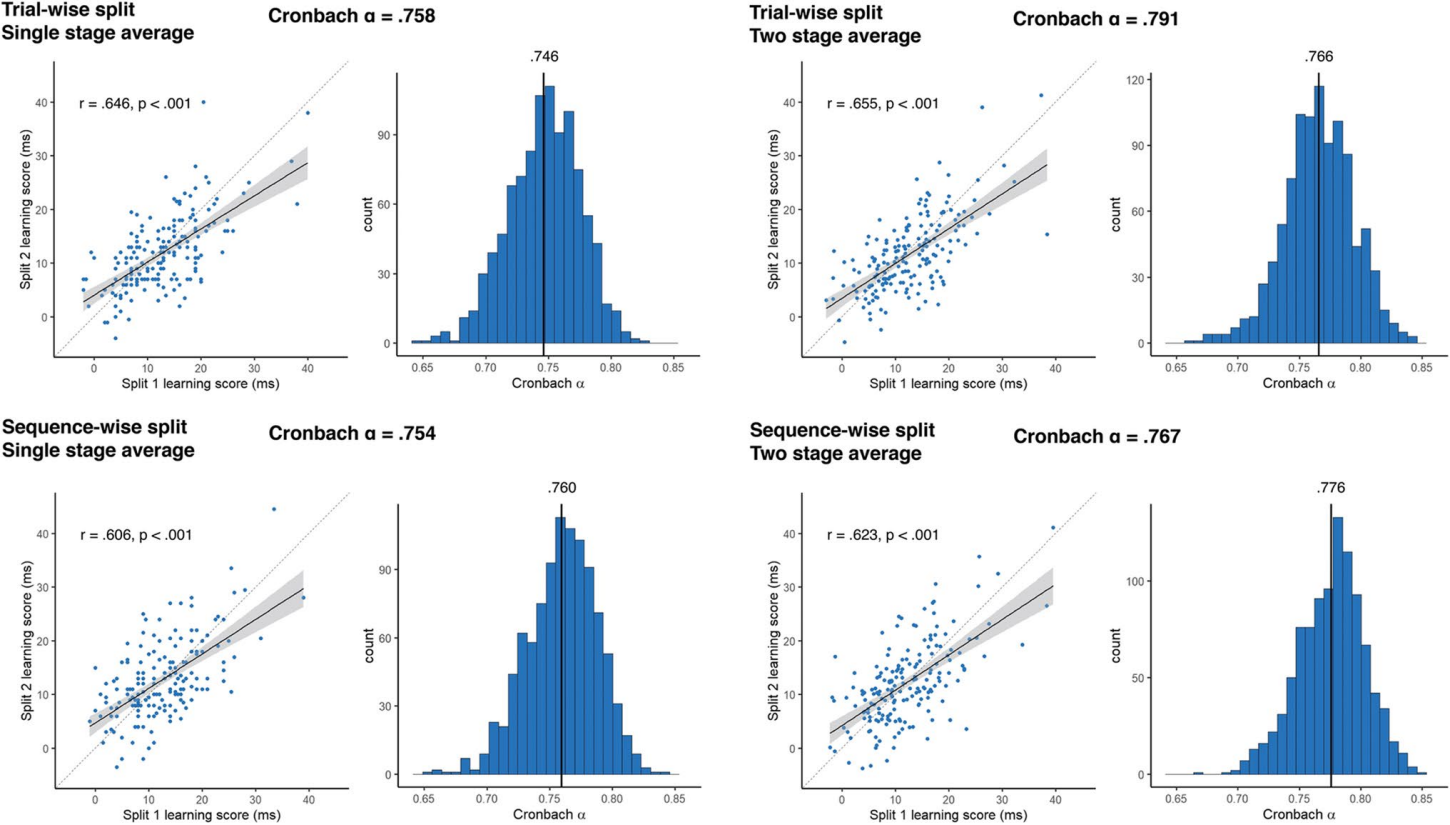
Reliability metrics for RT-derived learning scores. The four panels show the results of the four methods of reliability calculation that differ in pre-processing choices. In each panel, the Cronbach alpha on top of each panel shows the obtained alpha from the simple sequential assignment of trials, and its 95% CI calculated with Feldt's procedure. *Scatterplots* show learning scores the raw correlation between learning scores for the two splits, with one dot corresponding to one subject. Learning scores are in units of differences in reaction times for the two triplet types. The *trendline* shows linear fit, bands correspond to 95% CI. The *dashed line* shows the identity line. We also indicate the split-half Pearson's correlation and its *p* value, as well as 95% CI. Histograms show the results of the two permutation analyses, on the left, the distribution of Cronbach alphas resulting from trial resampling along with its mean, on the right, the bootstrapped distribution of Cronbach alphas, along with its mean, and the bootstrapped 95% CI values

**Correct Figure 2**

**Fig. 2:**
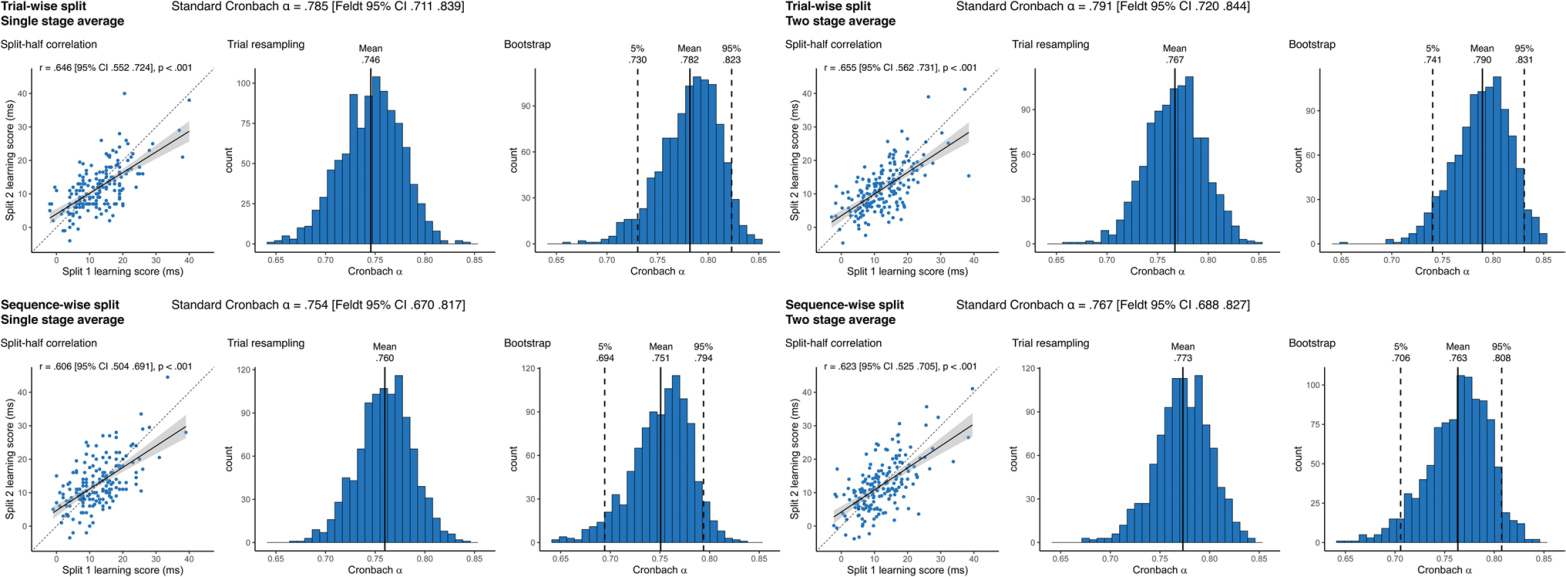
Reliability metrics for RT-derived learning scores. The four panels show the results of the four methods of reliability calculation that differ in pre-processing choices. In each panel, the Cronbach alpha on top of each panel shows the obtained alpha from the simple sequential assignment of trials, and its 95% CI calculated with Feldt's procedure. *Scatterplots* show learning scores the raw correlation between learning scores for the two splits, with one dot corresponding to one subject. Learning scores are in units of differences in reaction times for the two triplet types. The *trendline* shows linear fit, bands correspond to 95% CI. The *dashed line* shows the identity line. We also indicate the split-half Pearson's correlation and its *p* value, as well as 95% CI. Histograms show the results of the two permutation analyses, on the left, the distribution of Cronbach alphas resulting from trial resampling along with its mean, on the right, the bootstrapped distribution of Cronbach alphas, along with its mean, and the bootstrapped 95% CI values

**Incorrect Figure 3**

**Fig. 3:**
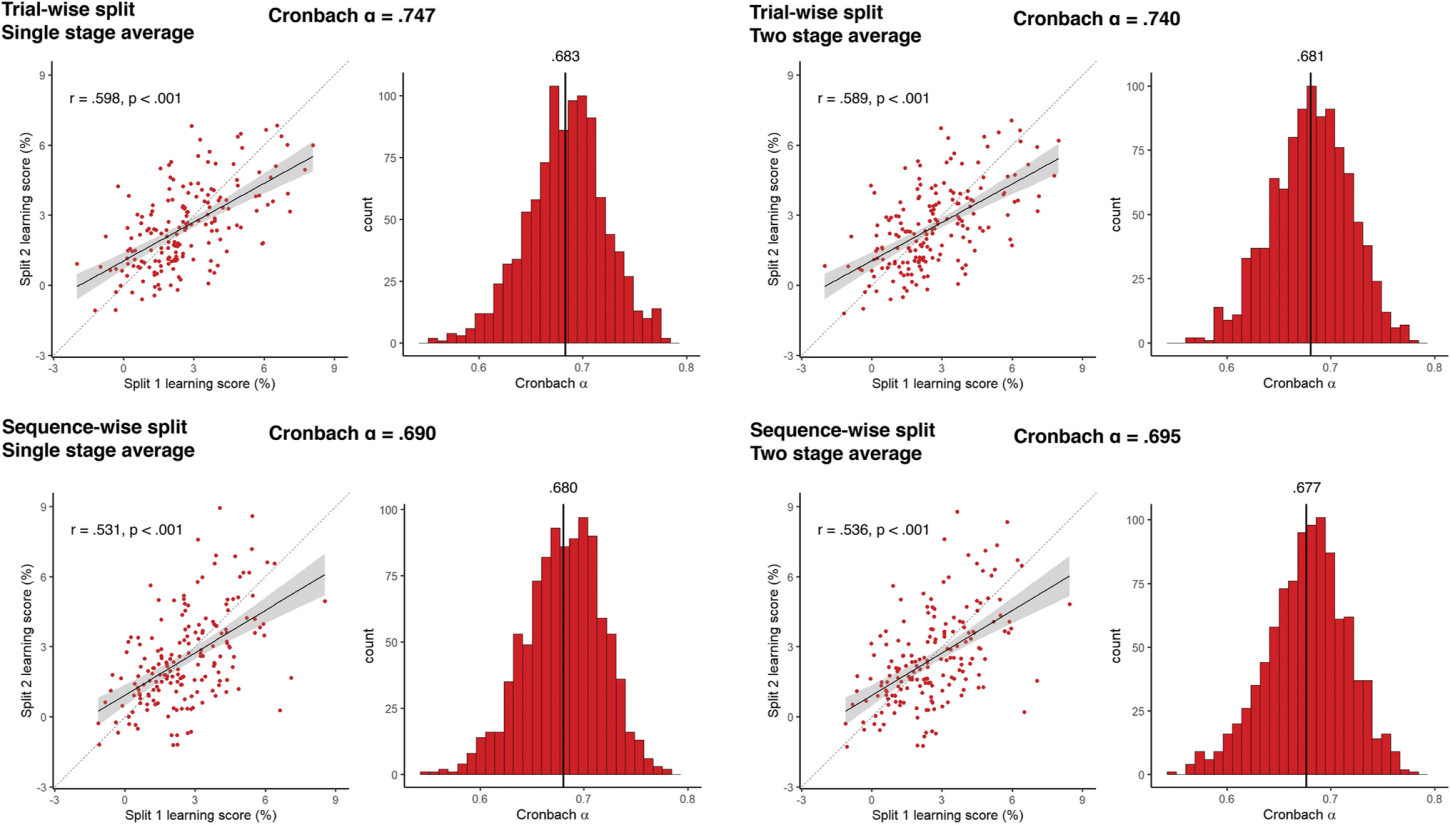
Reliability metrics for accuracy-derived learning scores. The four panels show the results of the four methods of reliability calculation that differ in pre-processing choices. In each panel the Cronbach alpha on top of each panel shows the obtained alpha from the simple sequential assignment of trials, and its 95% CI calculated with Feldt's procedure. *Scatterplots* show learning scores the raw correlation between learning scores for the two splits, one dot corresponding to one subject. Learning scores are in units of differences in reaction times for the two triplet types. The *trendline* shows linear fit, bands correspond to 95% CI. The *dashed line* shows the identity line. We also indicate the split-half Pearson's correlation and its *p* value, as well as 95% CI. Histograms show the results of the two permutation analyses, on the left, the distribution of Cronbach alphas resulting from trial resampling along with its mean, on the right, the bootstrapped distribution of Cronbach alphas, along with its mean, and the bootstrapped 95% CI values

**Correct Figure 3**

**Fig. 3:**
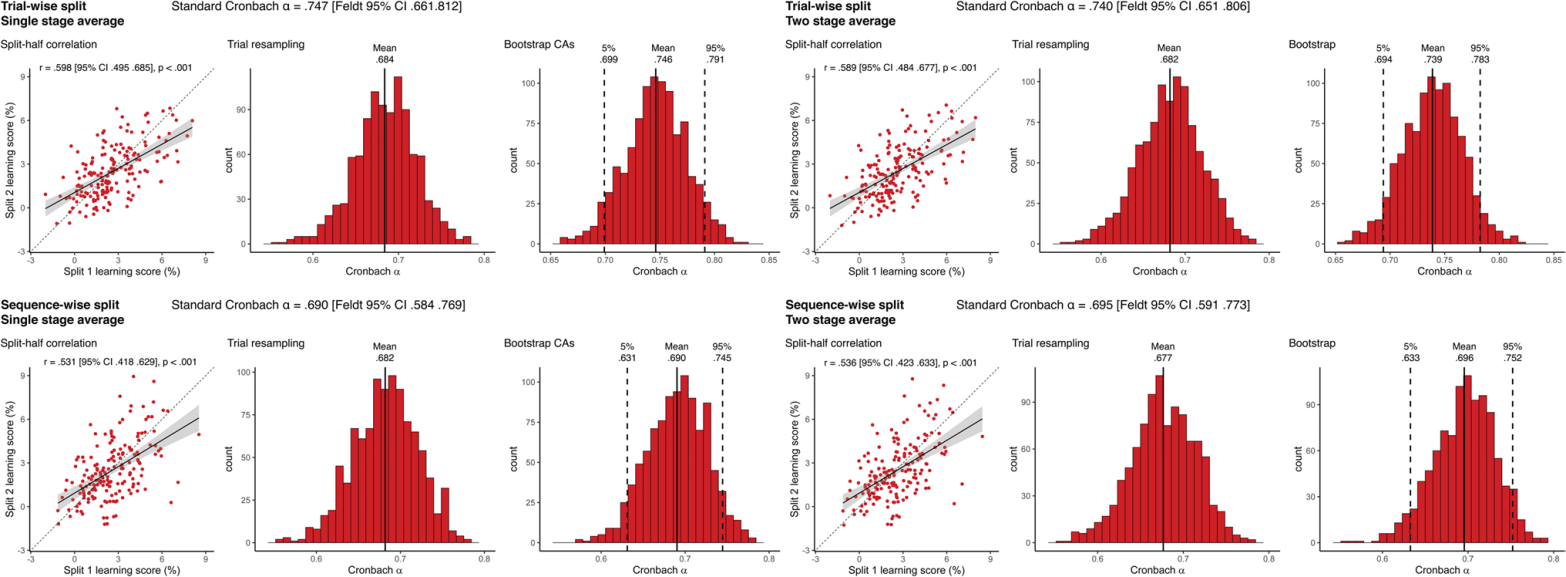
Reliability metrics for accuracy-derived learning scores. The four panels show the results of the four methods of reliability calculation that differ in pre-processing choices. In each panel the Cronbach alpha on top of each panel shows the obtained alpha from the simple sequential assignment of trials, and its 95% CI calculated with Feldt's procedure. *Scatterplots* show learning scores the raw correlation between learning scores for the two splits, one dot corresponding to one subject. Learning scores are in units of differences in reaction times for the two triplet types. The *trendline* shows linear fit, bands correspond to 95% CI. The *dashed line* shows the identity line. We also indicate the split-half Pearson's correlation and its *p* value, as well as 95% CI. Histograms show the results of the two permutation analyses, on the left, the distribution of Cronbach alphas resulting from trial resampling along with its mean, on the right, the bootstrapped distribution of Cronbach alphas, along with its mean, and the bootstrapped 95% CI values

